# Design and Manufacturing of a Passive Pressure Sensor Based on *LC* Resonance

**DOI:** 10.3390/mi7050087

**Published:** 2016-05-10

**Authors:** Cheng Zheng, Wei Li, An-Lin Li, Zhan Zhan, Ling-Yun Wang, Dao-Heng Sun

**Affiliations:** Department of Mechanical and Electrical Engineering, Xiamen University, Xiamen 361005, China; zhengcheng@stu.xmu.edu.cn (C.Z.); 19920141152871@stu.xmu.edu.cn (W.L.); lianlin@stu.xmu.edu.cn (A.-L.L.); snowkally@gmail.com (Z.Z.)

**Keywords:** pressure sensor, *LC* resonance, wireless

## Abstract

The *LC* resonator-based passive pressure sensor attracts much attention because it does not need a power source or lead wires between the sensing element and the readout system. This paper presents the design and manufacturing of a passive pressure sensor that contains a variable capacitor and a copper-electroplated planar inductor. The sensor is fabricated using silicon bulk micro-machining, electroplating, and anodic bonding technology. The finite element method is used to model the deflection of the silicon diaphragm and extract the capacitance change corresponding to the applied pressure. Within the measurement range from 5 to 100 kPa, the sensitivity of the sensor is 0.052 MHz/kPa, the linearity is 2.79%, and the hysteresis error is 0.2%. Compared with the sensitivity at 27 °C, the drop of output performance is 3.53% at 140 °C.

## 1. Introduction

Pressure sensors are widely used in areas such as industrial control, aerospace, environmental detection, and medical diagnosis. However, in the enclosed or harsh environments such as human bodies and engines, it is not economical or feasible to monitor pressure with a wired connection. Since the introduction of a battery reduces lifetime and reliability, a completely passive sensing element has more advantages. A passive pressure sensor based on *LC* resonance with variable resonant frequency was first made by COLLINS [1]. Afterward, more sensors adopting the same principle were fabricated with thick-film multilayer technology [2,3] or micro-electromechanical system technology [4,5,6]. Then, the application of *LC* sensors expanded to areas such as health care [5,7,8,9] and other areas with high-temperature environments [3,10]. Meanwhile, the design and optimization of the sensor have been partially revealed [11,12], and a readout system has also been developed [13].

In this paper, a passive pressure sensor based on *LC* resonance is realized with a parallel capacitor-inductor resonant circuit. The capacitor consists of two metal plates, and the capacitance changes due to varying pressure. The planar inductor is inside the sealed cavity of the sensor for easy connection to the sensitive capacitor. Therefore, it does not require the lead connection outside of the cavity, which simplifies the fabrication process. The sensor signal is transferred wirelessly by inductive coupling. Furthermore, the return loss *S*_11_ of the pressure system is measured to determine the sensor resonance frequency.

## 2. The Sensor Structure

**Figure 1:**
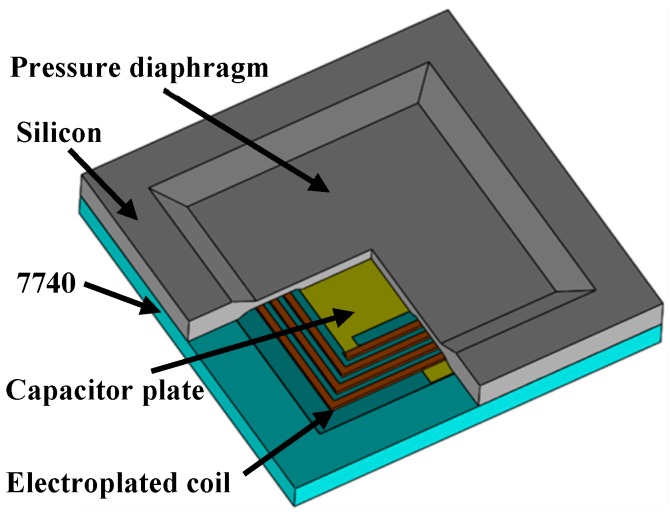
Structure of the passive pressure sensor.

The structure of the wireless pressure sensor is shown in Figure 1. The silicon and glass layers are bonded together to construct a vacuum-sealed cavity. The upper and lower metal plates are grown on silicon and glass separately inside the cavity to form the capacitor with a 6.5-μm gap. The 140-μm-thick pressure sensing diaphragm is achieved by etching the silicon. Thus, the capacitance varies when the applied pressure on diaphragm changes. The planar coil is fabricated on glass with two ends connected to the variable capacitor to form the *LC* resonator. The corresponding area on silicon is etched down to locate the coil. Unlike the 7740 glass, silicon is easier to be etched deeper without defects [14]. This is beneficial for fabricating thicker on-chip inductors with higher *Q*. Meanwhile, the 7740 glass substrate (Pyrex^®^, Corning Incorporated, Corning, New York, NY, USA, with *ε*_r_ = 4.6) of the inductor is used to decrease the parallel parasitic capacitance of the inductor [15] and to obtain high sensitivity for the sensor. The feature sizes of the inductor coil are shown in Table 1.

## 3. Measurement Principles

**Figure 2:**
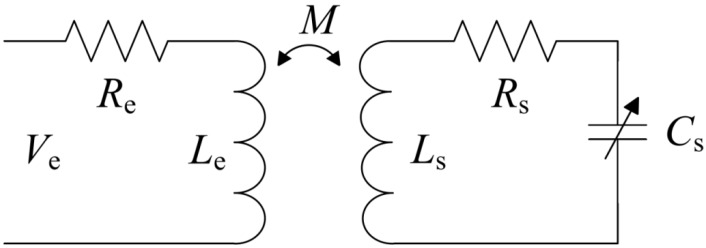
Equivalent circuit of the passive pressure sensor.

Figure 2 shows the equivalent circuit of the inductively coupled pressure sensor system [4]. The sensor is modeled with an inductor *L*_S_, series resistance *R*_S_, and a variable capacitor *C*_S_. The resonance frequency of the sensor is given by

(1) $$f_{0}=\frac{1}{2\pi \sqrt{L_{s}C_{s}}}\;$$

The quality factor of the sensor can be expressed as

(2) $$Q=\frac{\omega_{0}L_{s}}{R_{s}}=\frac{1}{R_{s}}\sqrt{\frac{L_{s}}{C_{s}}}$$

The equivalent input impedance *Z*_e_ of the readout coil is

(3) $$Z_{e}\left(f\right)=R_{e}+j2\pi fL_{e}\left(1+\left(\frac{k^{2}{\left(\frac{f}{f_{0}}\right)}^{2}}{1+j\frac{1}{Q}\frac{f}{f_{0}}-{\left(\frac{f}{f_{0}}\right)}^{2}}\right)\right),$$

where *R*_e_ is the series resistance of the reader coil, *f* is the frequency, and *k* is the coupling coefficient. The return loss *S*_11_ at terminals of the readout coil is

(4) $$S_{11}=\frac{Z_{e}\left(f\right)-Z_{0}}{Z_{e}\left(f\right)+Z_{0}}$$

The frequency where the magnitude of *S*_11_ reaches its minimum value is approximately equal to the resonant frequency *f*_0_.

## 4. The Simulation of the Pressure Diaphragm

**Figure 3:**
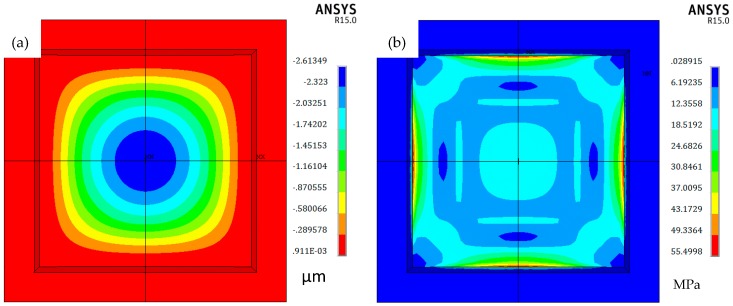
ANSYS simulation of diaphragm when *p* = 0.1 MPa. (**a**) Deflection; (**b**) Von Mises stress.

**Figure 4:**
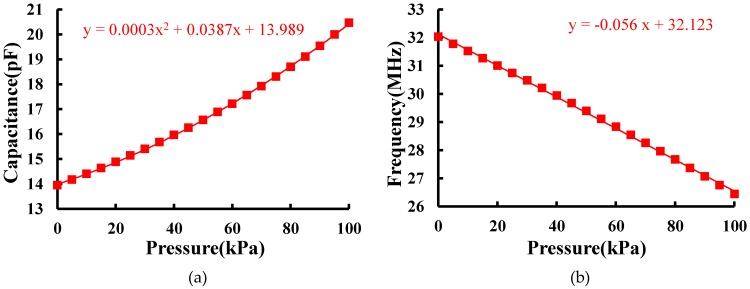
(**a**) Capacitance *vs*. pressure; (**b**) *f*_0_ *vs*. pressure in simulation.

FEM simulation of electromechanical analysis was conducted using ANSYS (Ansys, Inc., Cecil Township, PA, USA). In the simulation, the pressure ranges from 0 to 0.1 MPa. Figure 3 indicates that the deflection of the diaphragm is 2.6 μm when the applied pressure is 0.1 MPa, and the maximum von Mises stress at the edge of the membrane is 55.5 MPa, which is much lower than the yield point of monocrystalline silicon (7 GPa). The capacitance between two plates can be calculated by

(5) $$C=\frac{2E}{V^{2}}\;$$

in which *V* is the voltage between the two plates, and *E*, energy of the electrostatic field between the two plates of the capacitor, can be extracted from the simulation. Figure 4a shows that the capacitance varies with pressure ranges from 0 to 100 kPa, which fits a quadratic equation. The inductance of the coil is 1.8 μH calculated from the modified Wheeler formula described in [16]. Thus, the calculated *Q* is about 20, and the calculated *f*_0_ under pressure is shown in Figure 4b. The characteristic curve is approximately linear, and the analytical sensitivity is 0.056 MHz/kPa.

## 5. The Manufacturing Process

**Figure 5:**
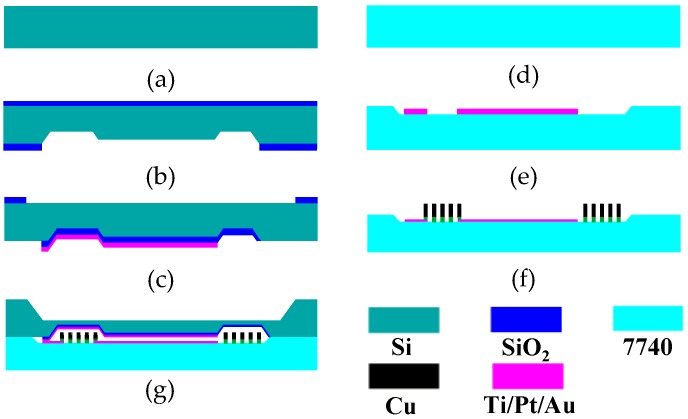
Fabrication process of the pressure sensor. (**a**) Silicon; (**b**) TMAH etch; (**c**) Upper plate sputter and pattern; (**d**) 7740 glass; (**e**) Lower plate sputter and pattern; (**f**) Planar coil electroplating; (**g**) Anodic bonding.

The passive pressure sensor is fabricated by the silicon bulk micromachining, electroplating, and anodic bonding technology. Figure 5 shows a flowchart of the manufacturing process. First, the oxide layer on the silicon is patterned with a Buffered oxide etch (BOE) solution. Then, a 55-μm recess and 6.5-μm capacitor gap are formed by wet etching using a 25% Tetramethylammonium hydroxide (TMAH) solution (Figure 5b). A 1.9-μm-thick oxide layer is patterned to be the insulation layer, and 500-nm Ti/Pt/Au is sputtered on the SiO_2_ layer as the upper plate (Figure 5c). After the lower plate of the capacitor is patterned using a lift-off process (Figure 5e), the planar coil is electroplated on the glass (Figure 5f) with patterned positive photoresist AZP4620 as the mold for electroplating. Although SU-8 is a popular negative photoresist used as an electroplating mold for its high aspect ratio and nearly vertical sidewalls, it is not chosen here due to the following disadvantages: great coating stress, low adhesion, resistance to residues after developing [17,18], and incompatibility with the acidic copper electroplating solution. The silicon and the glass are bonded together to form the *LC* resonator and the sealed cavity by anodic bonding. Finally, the silicon in bonded wafers is etched anisotropically to obtain the pressure diaphragm (Figure 5g).

**Figure 6:**
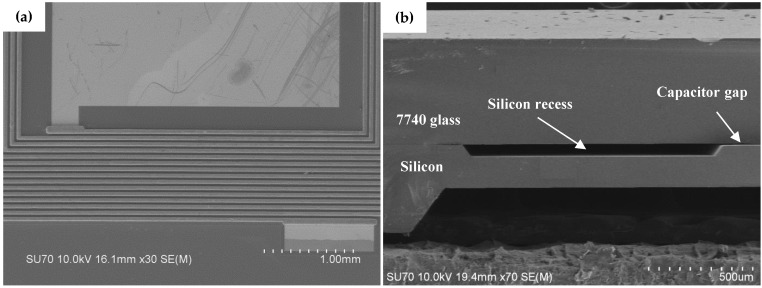
(**a**) SEM photograph of part of the coil and plate; (**b**) SEM photograph of a sensor showing the cross-sectional view.

Figure 6a shows a Scanning Electron Microscope (SEM) photograph of the copper-electroplated coil, and Figure 6b shows a cross section of the silicon diaphragm, silicon recess, and capacitive gap.

## 6. Test Results and Discussion

**Figure 7:**
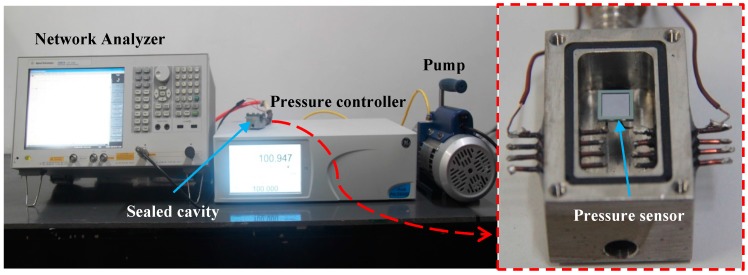
Frequency-pressure measurement system.

**Figure 8:**
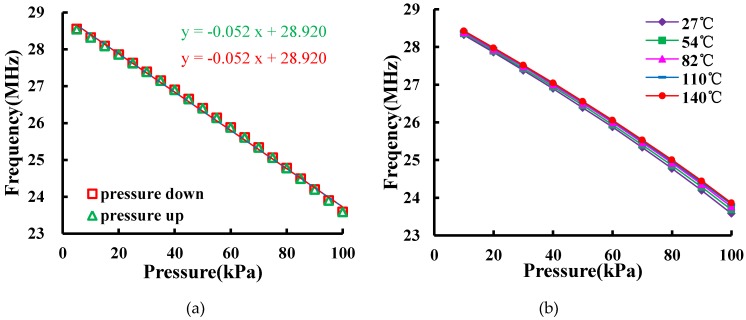
(**a**) Measured *f*_0_ *vs.* frequency; (**b**) measured *f*_0_ *vs.* frequency under different temperatures.

Figure 7 shows a frequency-pressure test system to measure the resonant frequency-pressure characteristic of the sensor. A Druck PACE6000 pressure controller (GE, Fairfield, CT, USA) connected to a vacuum pump is used to regulate the pressure in a sealed chamber. A 2.2-μH magnetic core winding inductor is served as the read coil. The sensor is fixed on top of the read coil without any electric connection inside the chamber. An Agilent E5061B network analyzer (Agilent Technologies, Santa Clara, CA, USA) is applied to measure *S*_11_. Figure 8a shows the measured *f*_0_ changes with the pressure fluctuating between 5 and 100 kPa. The result indicates that the sensitivity of the sensor is 0.052 MHz/kPa, the linearity *δ*_L_ is 2.79%, and the hysteresis error *δ*_H_ is 0.2%. The measured *f*_0_ is smaller than the calculated value. This is expected due to the existence of parasitic capacitance. Moreover, the fact that the fabricated diaphragm is thicker than the design value may lead to less sensitivity. The temperature testing result of the pressure sensor is plotted in Figure 8b. With the temperature varying from 27 to 140 °C, the drop of the sensitivity over temperature increments is 0.00185 MHz/kPa. Compared with the sensitivity at 27 °C, the drop of output performance is 3.53% at 140 °C. The temperature offset is caused by electromagnetic performance shift of the planar coil, the residual pressure in the sealed cavity, and the coefficients of thermal expansion between silicon and glass. 

## 7. Conclusions

A passive pressure sensor with a sealed *LC* circuit was designed and fabricated by a micro-electromechanical systems process. The experimental results show that the sensor has an almost linear signal output and an appreciable sensitivity. In addition, it is easy to alter the structure size to obtain optimal performance. The sensor can potentially be used on some occasions where wireless measurement is needed. Our future work will be directed towards increasing the sensor measurement distance range and extending the working temperature by optimizing the geometric parameters of the inductance coil and by utilizing silicon carbide as the bulk material, respectively.

## Figures and Tables

**Table 1:** The feature sizes of the coil.

Size Features	Value
Inner diameter of the inductor coil	3814 μm
Width of coil lines	30 μm
Spacing between coil lines	40 μm
Depth of inductor	27 μm
Number of inductor coil	14
